# Proceedings

**Published:** 1883-11

**Authors:** 


### PROCEEDINGS.

The Central Illinois Dental Society held its annual session in El Paso, Ill., on Oct. 10th and 11th. It was much the best session, held so far, by this young but vigorous society. In some respects it was even better than the sessions of the State Society. Clinical operations were carried on at five chairs, thus furnishing every one a good opportunity of witnessing the work at different stages of progress, without being crowded. Dr. J. M. Hurtt, of Peoria, restored the corner of a central incisor, using gold screws. Dr. E. B. Call, of Peoria, made and placed a gold crown on a bicuspid for a lady. Dr. E. D. Swain, of Chicago, put in a central incisor by the Richmond method, and also with the Swain crown. Dr. C. R. Taylor, of Streator, restored point of cuspid, using gold and platina foil. Dr. A. W. Harlan, of Chicago, gave a clinic on treatment of alveolitis, which was especially helpful to many dentists present. The papers read were as follows:

President's Address—Dr. G. Newkirk, Chicago; Theories, Dr. W. A. Johnson, Peoria; Dentistry and its Present Status, Dr. K. B. Davis, Springfield; Bacteria, Dr. E. D. Swain, Chicago; The Higher Education, Dr. J. D. Moody, Mendota; Alveolitis Dr. A. W. Harlan, Chicago; Robinson's Metal as a Filling Material, Dr. T. W. Brophy, Chicago; Filling Root Canals, Dr. C. W. Spaulding, St. Louis Prenatal Influences as Affecting the Teeth. Dr. Geo. H. Cushing, Chicago.

Dr. Harlan's paper, with the discussion which followed, and the discussion of Dr. Spaulding's paper, on Wednesday evening, were of real practical benefit to all. The papers of Drs. Swain and Cushing furnished abundant food for the more thoughtful.

"Incidents of Office Practice," Tuesday evening, was participated in by a large number, and was very interesting. Altogether it was a very enjoyable and profitable meeting. M.

